# Supplementary material for: Single-cell isotope tracing reveals functional guilds of bacteria associated with the diatom Phaeodactylum tricornutum
Source: Nat Commun. 2023 Sep 13;14:5642. doi: 10.1038/s41467-023-41179-9 (PMC10499878; doi:10.1038/s41467-023-41179-9)
Supplement: Supplementary file 1 — Supplementary Information [file 41467_2023_41179_MOESM1_ESM.pdf]

Supplementary information for "Single-cell isotope tracing reveals functional guilds of bacteria associated with the diatom *Phaeodactylum tricornutum*"

Supplementary genomics and proteomics results

Here we present further results of the genomic analysis of the 15 bacterial strains associated with *P. tricornutum* laboratory batch cultures (see supplementary data 1 for links to the raw data and genome annotations). We found no evidence of de-novo net carbon fixation, except that *Devosia* showed capacity for carbon fixation via the 3-hydroxypropionate bi-cycle (supplementary data 3), which remains to be tested experimentally. None of the strains appear to be capable of methanogenesis where methane is produced via anaerobic respiration, as all lack the two possible pathways. However, 5 strains may be able to produce methane aerobically as a byproduct during conversion of methylphosphonate in the C-P lyase pathway (supplementary data 3). None of the strains appear to be methanotrophs, as all are lacking the pathway to convert methane to formaldehyde. Four strains appear to be capable of light harvesting mechanisms, either through proteorhodopsin-based phototrophy (*Algoriphagus* and *Muricauda*) or the light-harvesting system II of aerobic anoxygenic phototrophy (AAP; *Roseibium* and *Yoonia*; (supplementary data 3).

We next examined both synthesis and degradation of carbohydrates, which are major components of algal organic matter, both extracellular and serving as intracellular storage compounds, the latter primarily as chrysolaminarin in the case of *P. tricornutum*. All bacterial strains demonstrate the capabilities to metabolize carbohydrates via at minimum one of five

pathways: Embden-Meyerhof-Parnas (EMP), phosphorylative or semi-phosphorylative Entner-Doudoroff, or pentose phosphate pathway (oxidative or non-oxidative; supplementary data 3). Of the five carbohydrate metabolism pathways, only the non-oxidative pentose phosphate pathway was identified across all genomes. An average of at least 3 carbohydrate metabolism pathways ($\bar{x}$ = 3.26) were complete. The maximum number of complete carbohydrate degradation pathways that a genome contained was 5, with *Algoriphagus*, *Arenibacter*, *Yoonia*, and *Rhodophyticola* demonstrating the most diverse metabolic capacities for carbohydrate metabolism (supplementary data 3). *Pusillimonas*, *Oceanicaulis*, and *Henriciella* contained the fewest complete pathways for carbohydrate metabolism, with just one complete pathway. Regarding carbohydrate synthesis, the glyoxylate cycle, a modified TCA cycle for biosynthesis of carbohydrates, contains 9 pathways for formation of metabolic intermediates. Twelve of the 15 bacterial genomes contain at least one complete of the 9 pathways, with *Rhodophyticola* encoding the greatest number of pathways (n = 7/9 pathways), and *Muricauda* and *Pusillimonas* lacking any complete pathways within the glyoxylate cycle. Eight of 9 pathways in the cycle were identified in at least one bacterial strain, with just the glyoxylate to pyruvate pathway lacking or incomplete from all genomes. The most conserved pathways across the 15 strains are the phosphoglycolate to glyoxylate (n = 10 genomes) and the ethylmalonoyl-CoA (lower – succinyl-CoA; n = 8 genomes; supplementary data 3). Five strains are capable of oxidizing glycolate, a product of algal photorespiration (*Yoonia*, *Marinobacter*, *Stappia*, *Thalassospira*, and *Rhodophyticola*), all of which except *Rhodophyticola* incorporated it empirically, but surprisingly fewer strains have annotated genes for transporting glycolate across the cell membrane using glycolate permease (n = 3 strains: *Alcanivorax*, *Thalassospira*, *Rhodophyticola*). This suggest that glycolate transporters are not well identified in the databases.

We also examined the potential for synthesis and catalysis of components outside of central carbon metabolism that have been previously implicated in mutualistic or antagonistic interactions with microalgae, but that we did not test empirically in this study. In addition to being produced by plants and algae (including *P. tricornutum*), phenylacetic acid (PAA) and indole-3-acetic acid (IAA) are plant growth-promoting substrates produced by some groups of bacteria. Ten of the strains are capable of phenylacetic acid (PAA) catabolism (n = 10), with two (*Alcanivorax* and *Algoriphagus*) also capable of synthesizing indole-3-acetic acid (IAA) from tryptophan, which they can accomplish using just one of the five potential pathways via amino acid decarboxylase (supplementary data 3). Another algal compound of interest, *p*-coumaric acid (pCA), is often produced during algal senescence and inhibits bacteria (Lou et al 2012). Seven of the strains can degrade pCA: *Devosia*, *Roseibium*, *Stappia*, *Yoonia*, *Rhodophyticola*, *Thalassospira*, and *Pusillimonas* (supplementary data 3). This compound is known to stimulate production of algicidal roseobacticides, a class of tropodithietic acid (TDA) produced by the Roseobacter clade, but none of the bacterial genomes, including the 4 isolates belonging to Rhodobacteraceae, contain the metabolic pathways for TDA/roseobacticide synthesis. Two bacterial isolates were found to additionally tolerate antimicrobial pCA via the intermembrane phospholipid transport system for maintaining membrane integrity (Calero et al 2018), including *Marinobacter* and the pCa-degrading *Thalassospira* (supplementary data 3). The two Gammaproteobacteria (*Alcanivorax* and *Marinobacter*), as well as an Alphaproteobacterium (*Thalassospira*), contain the genomic capability for the Gammaproteobacteria-specific Gac/Rsm density-dependent secondary metabolism and carbon storage regulation system (Ferreiro and Gallegos 2021), a regulatory pathway involved in signal transduction, suggesting that these taxa

can communicate with bacterial cohorts (e.g. quorum sensing, biofilm formation) or perform biochemical signaling with algae.

We also carried out an extensive analysis of the vitamin production capability of the strains, since vitamin exchange is one of the identified mechanisms of algal-bacterial mutualism. Note that the *F*/2 algal medium contains vitamins B1, B7, and B12. Eleven of the 15 isolates are capable of synthesizing at least one class of B vitamins, with five (*Alcanivorax, Arenibacter, Marinobacter, Pusillimonas, Tepidicaulis*) capable of synthesizing vitamin B7 (biotin) and seven containing at least one complete of three pathways for B12 synthesis (*Alcanivorax*, *Devosia*, *Roseibium*, *Yoonia*, *Stappia*, *Thalassospira*, and *Rhodophyticola*; (supplementary data 3). None of the bacterial strains can synthesize vitamin B1 (thiamine). Vitamin synthesis of B12 and B7 had no overlap, meaning that if a strain could synthesize B12, it could not synthesize B7, and vice-versa (supplementary data 3).

The main manuscript discusses the transport proteins expressed by *Rhodophyticola* and *Marinobacter* during co-cultivation with *P. tricornutum*, and here we briefly discuss other proteomics results. We detected a large number of proteins involved in protein synthesis, transport and energy precursor generation in both taxa (Fig. S12). We also detected outer membrane proteins in both taxa, and *Marinobacter* expressed outer membrane proteins related to surface attachment (categorized in either outer membrane/cell envelope or motility chemotaxis, Fig. 3A), in agreement with observing *Marinobacter* occasionally attached to *P. tricornutum* cells. Unexpectedly since these strains have been in culture with the diatom for many generations, we found that these bacteria expressed genes related to oxidative stress or energy

starvation. *Marinobacter*, for example, highly expressed a greater percentage of stress response proteins than *Rhodophyticola*, many of which were oxidative stress response proteins, such as superoxide dismutase and catalase-peroxidase KatG, the latter previously associated with bacterial mutualism with a picocyanobacterium (Hennon et al 2018). Under the energy and central metabolism category, both bacteria expressed genes for common pathways such as the TCA cycle, glycolysis, and pyruvate metabolism, but *Rhodophyticola* also highly expressed three syntenic aerobic carbon monoxide (CO) dehydrogenase proteins (supplementary data 2), suggesting oxidization of CO for energy during co-cultivation, which is generally thought to occur under conditions of low organic nutrients (Cordero et al 2019). We note that out of the 15 strains, 6 encode this CO oxidation pathway (supplementary data 3), suggesting this is a common metabolism in co-culture with algae, though measurements of the amount of C processed via CO oxidation are currently lacking. In *Marinobacter*, we detected the narG protein that codes for respiratory nitrate reductase (IMG locus tag Ga0264245_1341; (supplementary data 2), but it was only found in one technical replicate of one of the five biological replicates, so it did not pass our significance threshold. However, it suggests that *Marinobacter* may carry out low levels of denitrification for energy generation, which could contribute to its ability to grow in artificial seawater without the diatom.

Additional References

1. Calero P, Jensen SI, Bojanovič K, Lennen RM, Koza A, Nielsen AT (2018). Genome-wide identification of tolerance mechanisms toward p-coumaric acid in Pseudomonas putida. *Biotechnology and Bioengineering* **115:** 762-774.

2. Cordero PRF, Bayly K, Man Leung P, Huang C, Islam ZF, Schittenhelm RB *et al* (2019). Atmospheric carbon monoxide oxidation is a widespread mechanism supporting microbial survival. *The ISME Journal* **13:** 2868-2881.

3. Ferreiro MD, Gallegos MT (2021). Distinctive features of the Gac-Rsm pathway in plant-associated Pseudomonas. *Environmental Microbiology*.

4. Hennon GMM, Morris JJ, Haley ST, Zinser ER, Durrant AR, Entwistle E *et al* (2018). The impact of elevated CO2 on Prochlorococcus and microbial interactions with 'helper' bacterium Alteromonas. *The ISME Journal* **12:** 520-531.

5. Lou Z, Wang H, Rao S, Sun J, Ma C, Li J (2012). p-Coumaric acid kills bacteria through dual damage mechanisms. *Food Control* **25:** 550-554.

Supplementary Figures

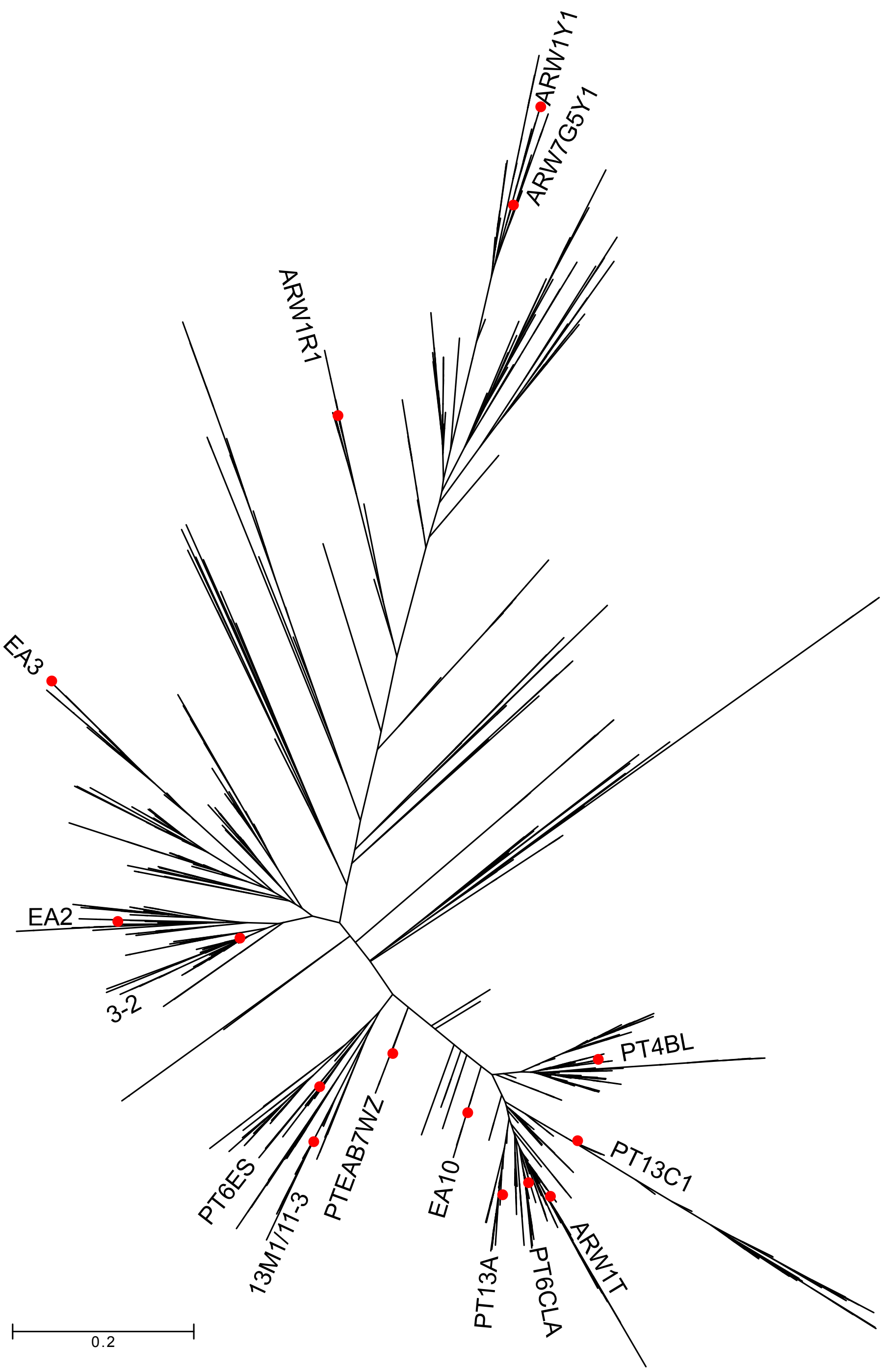

Figure S1: Phylogenetic tree of 1,443 16S rRNA gene amplified sequence variants from P. tricornutum enrichment cultures from Kimbrel et al. 2019, with the branch tips for the 15 isolates added and highlighted in red.

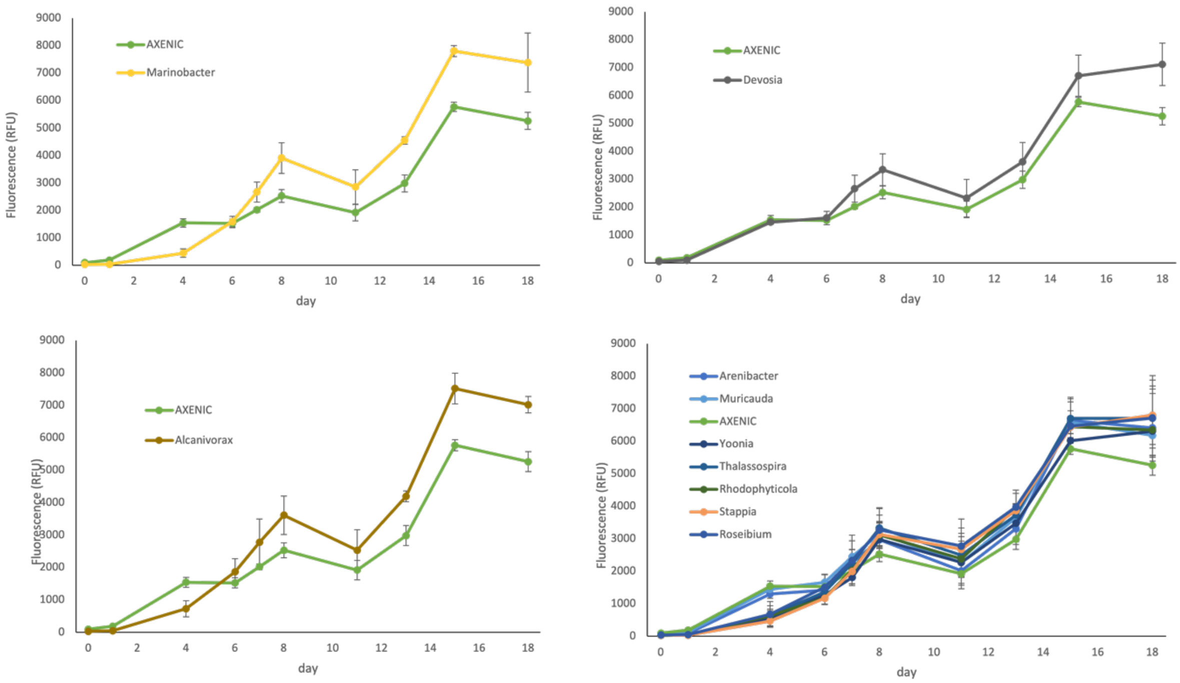

Figure S2 Growth of P. tricornutum co-cultured with single bacteria monitored by in-vivo chlorophyll fluorescence, showing increased fluorescence at day 18 compared to axenic cultures (200 microliter volume, mean +/- standard deviation; n = 3 cultures for each data point). Also shown are 7 other co-cultures showing equivalent fluorescence values to the axenic (not statistically different). Note that early phase (prior to day 6) shows a different pattern, where the following co-cultures showed a growth delay compared to axenic: Alcanivorax, Marinobacter, Stappia, Algoriphagus, Roseibium, Rhodophyticola, Thalassospira, and Yoonia.

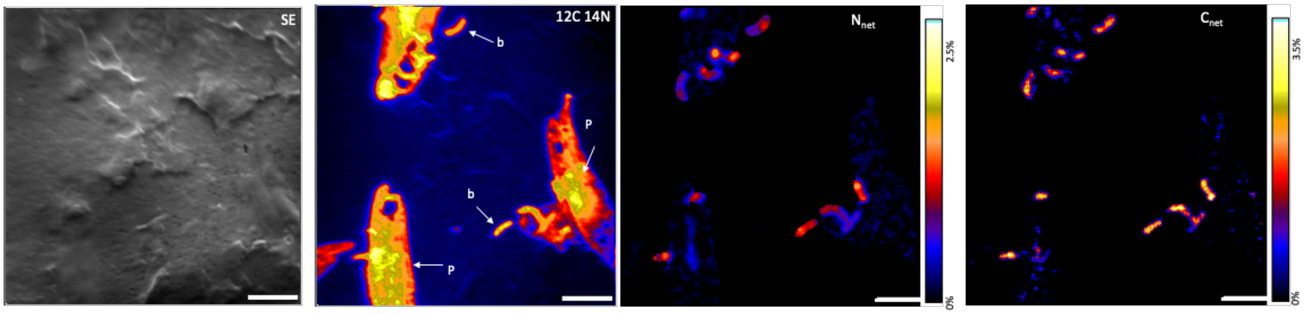

Figure S3: representative NanoSIMS images (secondary electron, SE, $^{12}C\ ^{14}N^-$, and ^{15}N and ^{13}C enrichment) showing P. tricornutum (P) and bacteria (b) incubated with ^{15}N and ^{13}C labeled algal organic matter (scale bar = 5 microns)

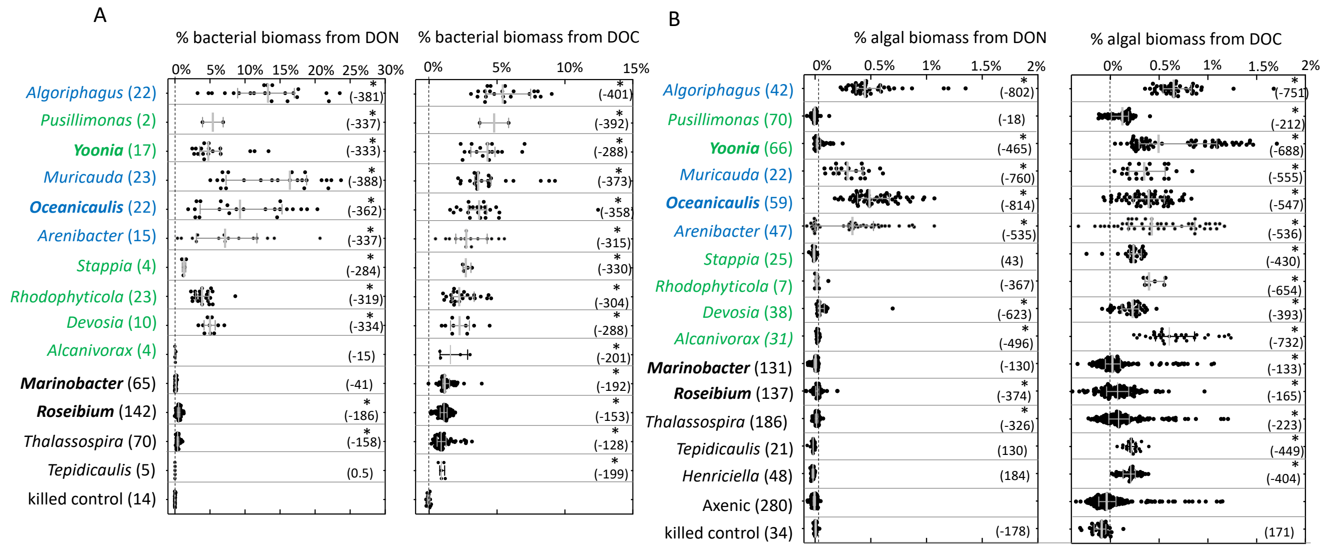

Figure S4: daily percentage of C and N biomass from algal Dissolved Organic Carbon (DOC) and Dissolved Organic Nitrogen (DON) incorporated (A) by single bacteria (median +/- 95% C.I.; n = 425 cells) and (B) by single algae as measured by ^{13}C and ^{15}N incorporation (median +/- 95% C.I.; n = 930 cells). Asterisks show cultures significantly more enriched than killed controls (mean rank difference shown in parentheses) using a two-sided non-parametric Kruskal-Wallis test with p-value adjustment for multiple comparisons with Dunn's method; bold name indicates this bacterium can attach to diatom cells)

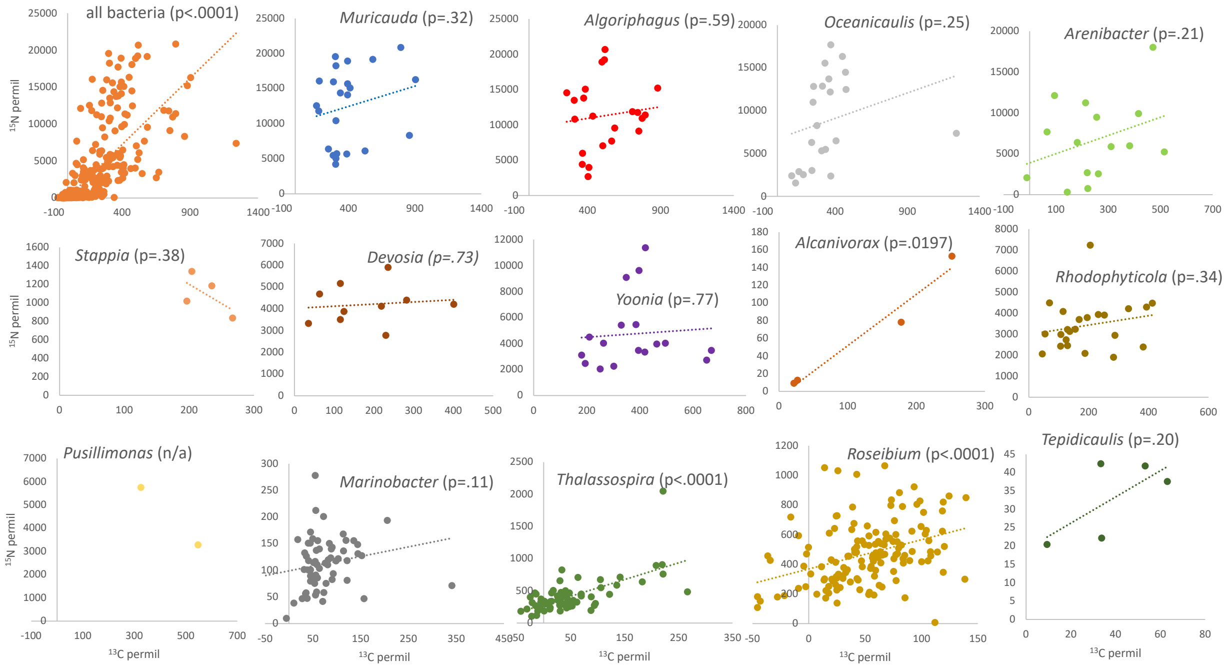

Figure S5: cell-specific nanoSIMS data showing relationship between ^{13}C and ^{15}N enrichment (reported in permil), showing certain strains with coupled Dissolved Organic Carbon (DOC) and Dissolved Organic Nitrogen (DON) incorporation (statistically significant based on linear regression) and others showing uncoupled DOC and DON incorporation (ns = not significant).

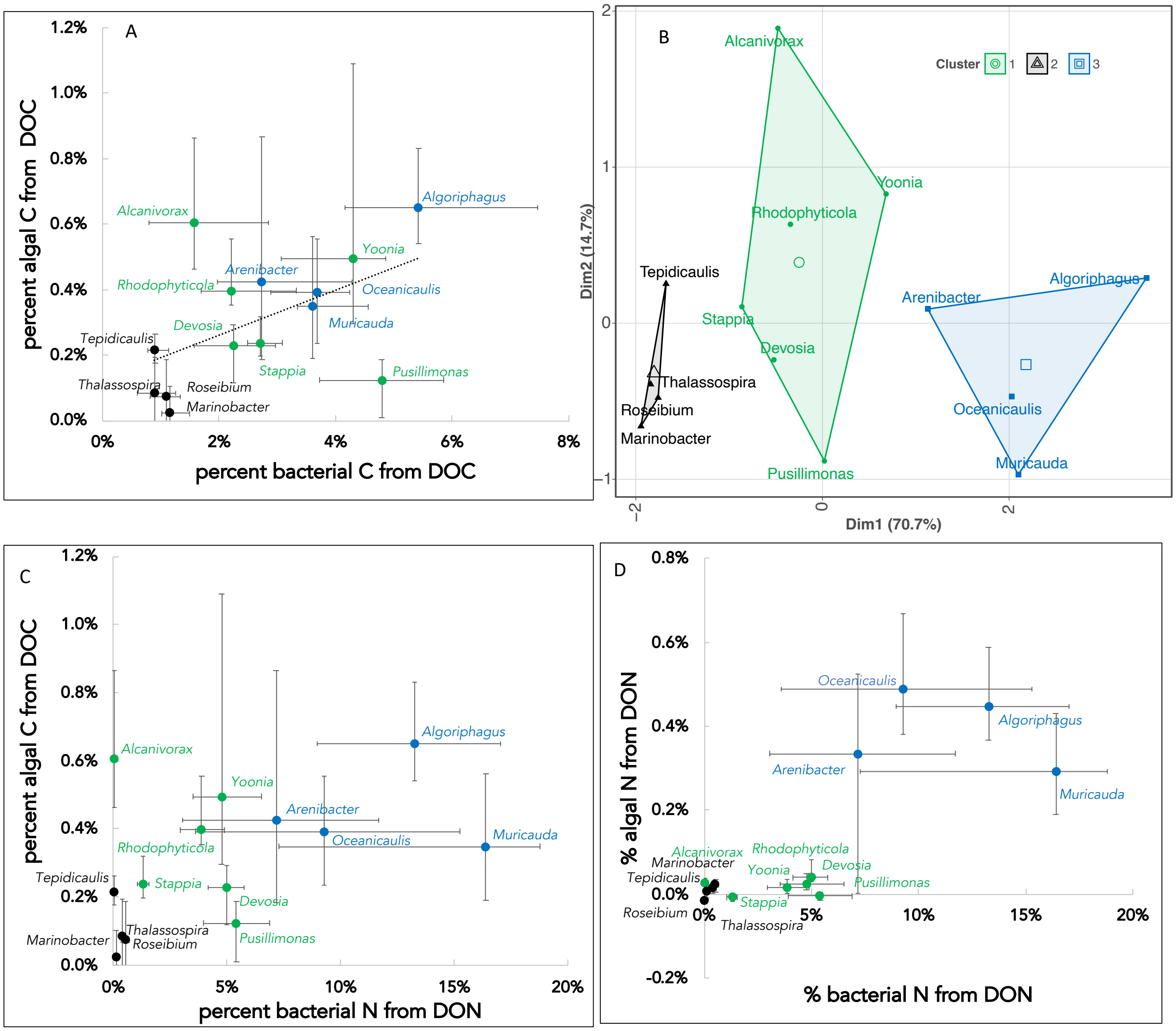

Figure S6: NanoSIMS data summarized by strain, showing median Dissolved Organic Carbon (DOC) and Dissolved Organic Nitrogen (DON) incorporation +/- interquartile range of (A) bacterial C and algal C; the C and N data were used for a K-means clustering analysis (B), which distinguished the 15 strains into 3 categories; using measured data, these 3 categories were best separated when plotting mean N incorporated by bacteria vs C remineralized to algae (median +/- interquartile range; C). Also shown is DON incorporated by bacteria versus N remineralized to algae (median +/- interquartile range; D). The total number of cells used for analysis were 425 for the bacteria and 930 for the algae.

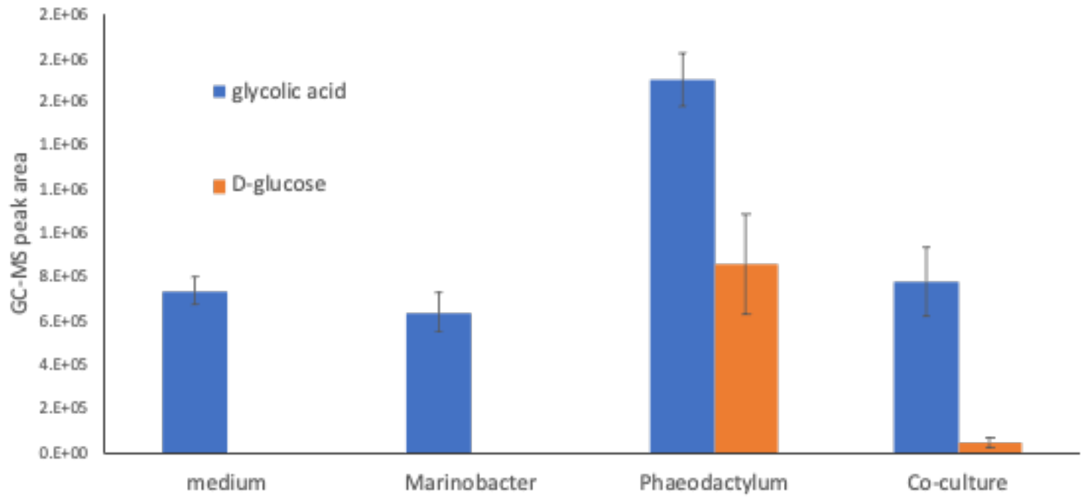

*Figure S7: Gas Chromatography-Mass Spectrometry *GC-MS) detection of 2 small polar metabolites in axenic P. tricornutum, axenic Marinobacter, and co-cultures compared to media blank (mean +/- standard error; n = 12 independent cultures)*

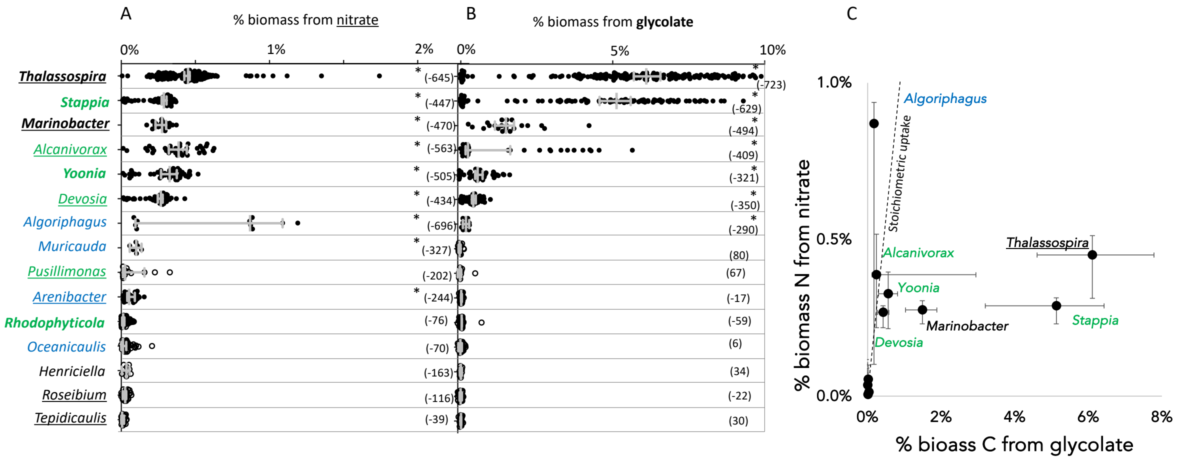

Figure S8 Bacterial incorporation of nitrate and glycolate measured by stable isotope incubations followed by nanoSIMS analysis. (A) fraction of C biomass from nitrate incorporated by single bacterial cells (median +/- 95% C.I.; 783 total cells) as measured by ^{15}N incorporation (asterisks indicate cultures significantly more enriched than killed controls using a two-sided non-parametric Kruskal-Wallis test with p-value adjustment for multiple comparisons with Dunn's method; in parentheses are the reported mean rank differences); (B) fraction of C biomass from glycolate incorporated by bacterial cells (median +/- 95% C.I.; n = 783 cells) as measured by ^{13}C incorporation (asterisks indicate significantly enriched compared to killed controls using a two-sided non-parametric Kruskal-Wallis test with p-value adjustment for multiple comparisons with Dunn's method; mean rank differences are shown in parentheses); (C) relationship between C and N incorporation for the bacterial populations (median +/- error bars representing 25% and 75% confidence intervals); genus names in bold indicate genomic capability of glycolate incorporation, and underlined indicate capability of assimilatory nitrate reduction; (n = 783 cells).

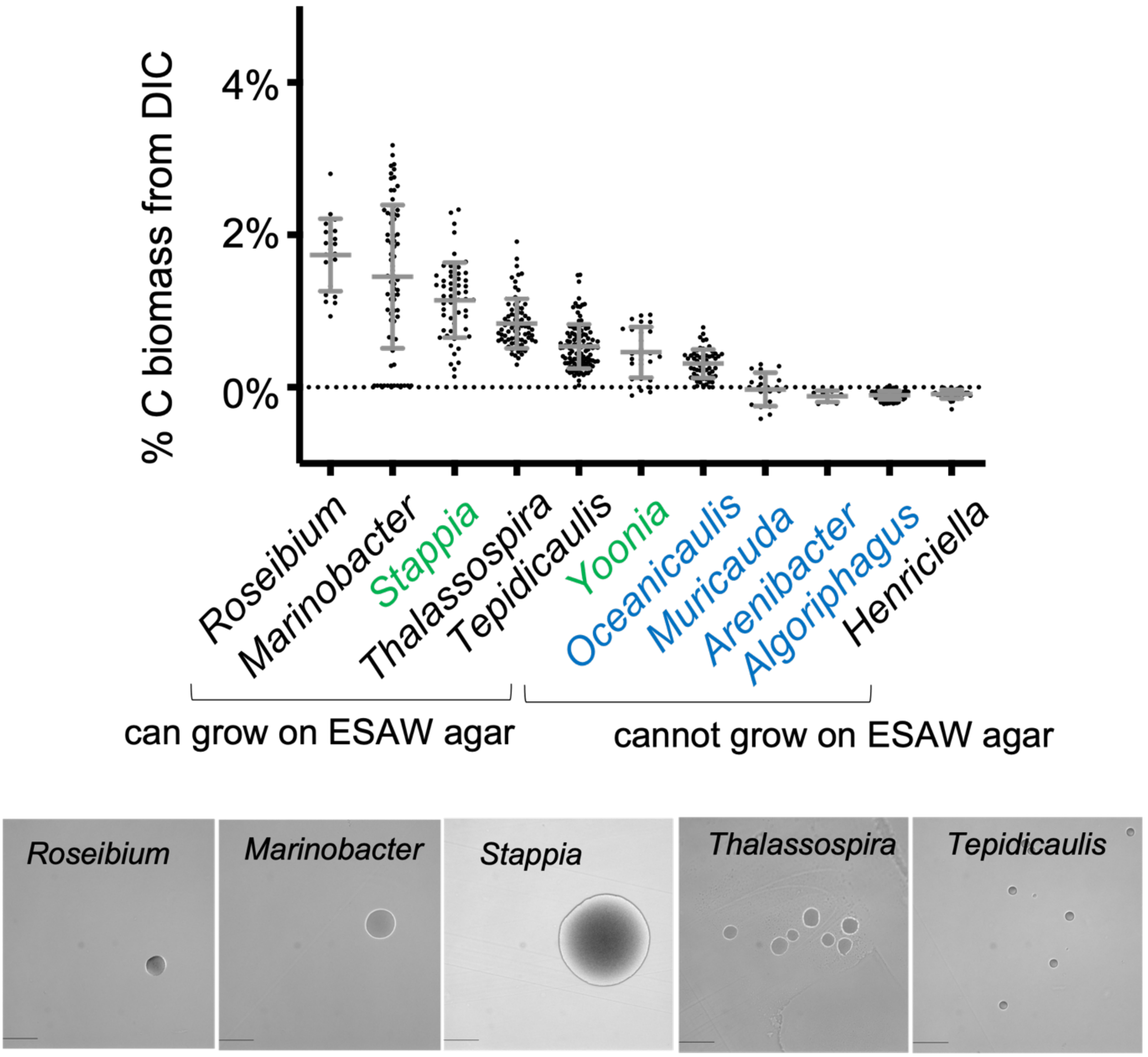

Figure S9: Net C biomass percent from dissolved inorganic carbon (DIC) incorporated over 1 week by 11 bacteria grown in artificial seawater medium (ESAW). The 5 strains on the left can grow on ESAW-agar (see colonies below; scale bar = 0.1 mm), and the 6 strains on the right cannot. Colors represent the 3 functional groups identified by K-means clustering of DOC and DON incorporation and remineralization (mean +/- standard deviation; n = 610 cells).

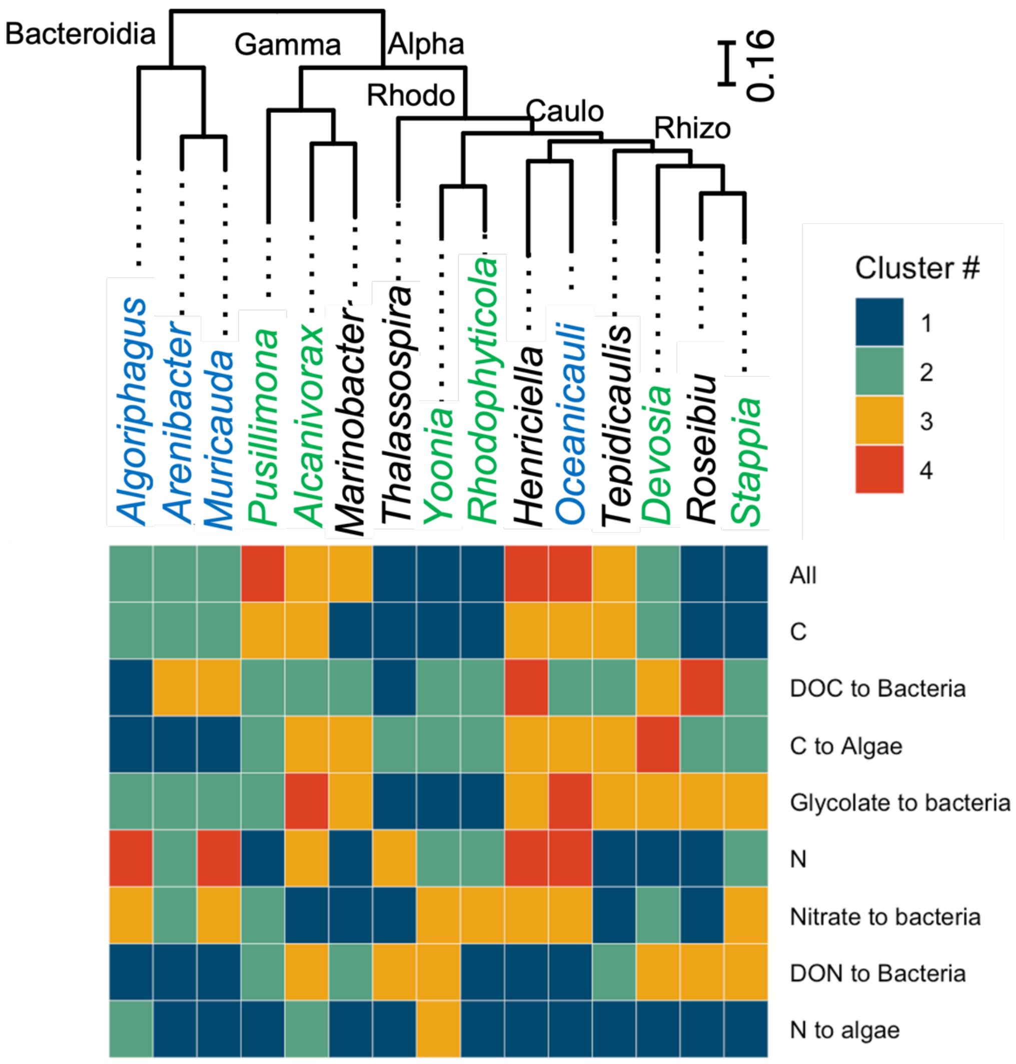

Figure S10: Cluster analysis based on the presence of metabolic pathways in the genomes of the 15 strains mapped onto the 16S phylogeny. Clusters were generated based on presence of genes related to C or N metabolism, nitrate or glycolate metabolism, N remineralization, organic C and N incorporation, and the combination of all. None of the clusters correspond to either phylogeny (based on the tree) or nanoSIMS activity (based on the color of the strains). Abbreviations: Rhizo = Rhizobacterales, Caulo – Caulbacterales, Gamma = Gammaproteobacteria, Rhodo = Rhodobacterales, Alpha = Alphaproteobacteria; DOC = Dissolved Organic Carbon, DON = Dissolved Organic Nitrogen.

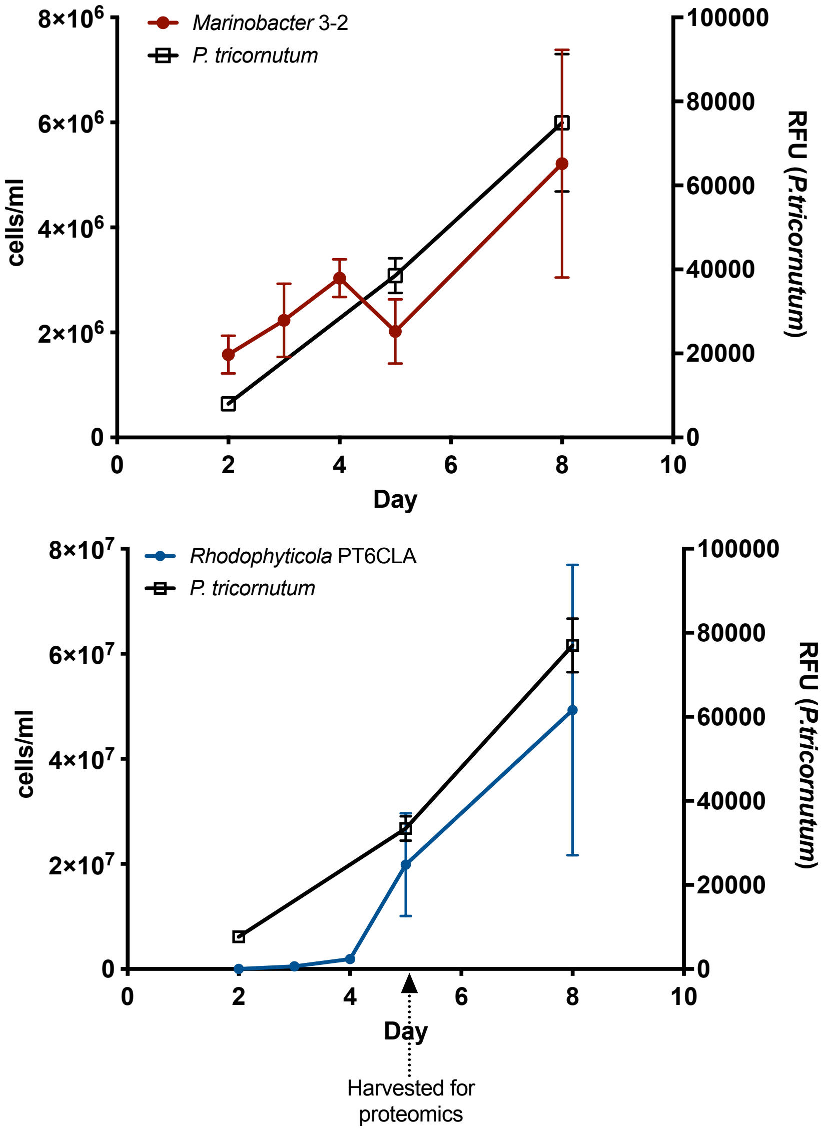

Figure S11: mean counts (+/- standard deviation) of DAPI stained bacterial cells and Relative chlorophyll Fluorescence Units (RFU) of P. tricornutum over 8-day co-cultivation, indicating harvest for proteomics (n = 6 independent flasks)

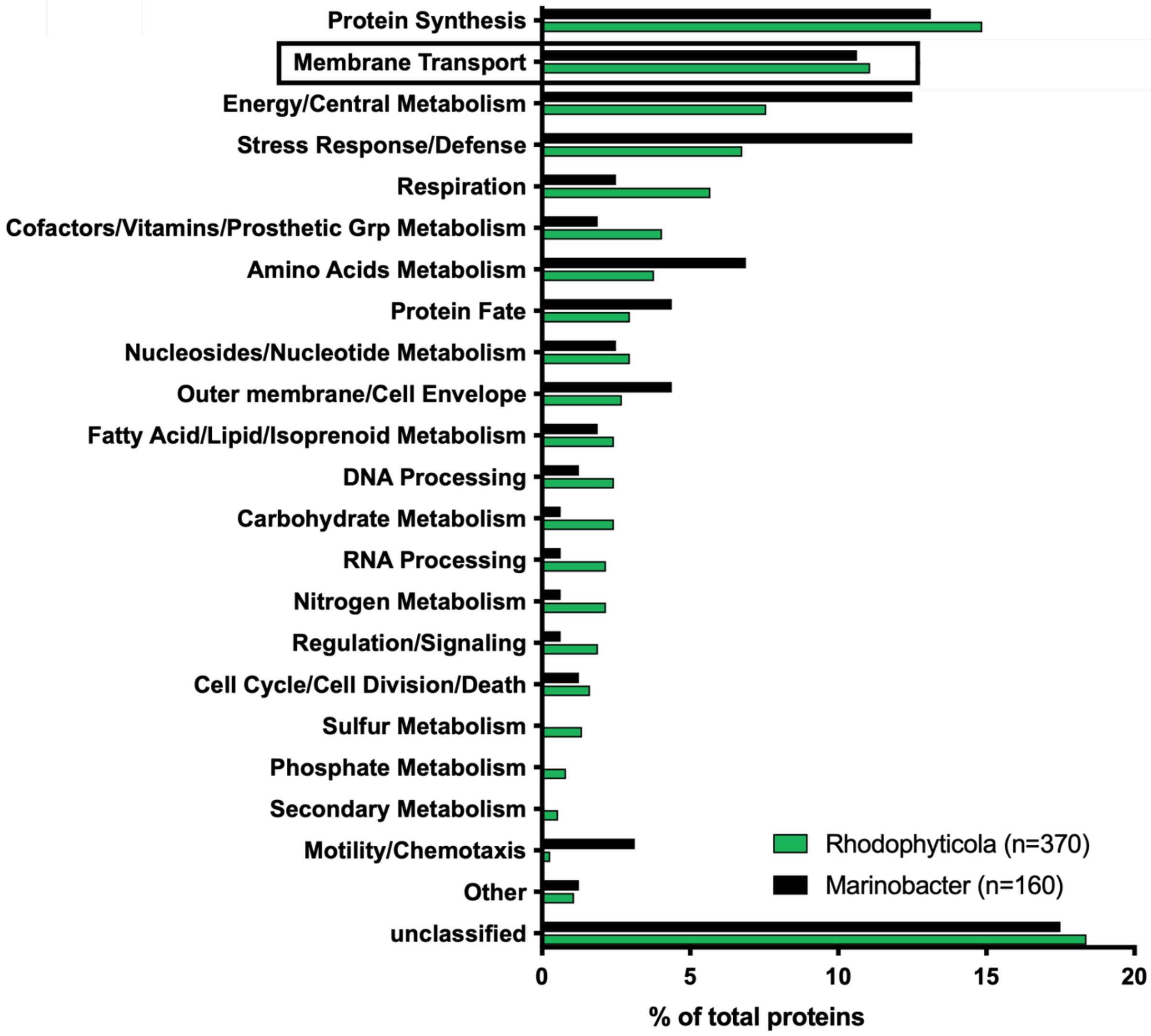

Figure S12: Summary of proteomics from Rhodophyticola and Marinobacter when grown in co-culture with P. tricornutum, representing the total percentage of proteins detected as annotated from the categories. Categories based on NCBI's PATRIC (Pathosystems Resource Integration Center) functional classifications to the "Superclass/class" level, including some manual additions to the categories (see supplemental information). "Other" category includes proteins that were less than 1% of the total proteins in both taxa, and include functional classes "clustering based subsystems", "Iron acquisition and metabolism", "Metabolite damage and its repair" and "Miscellaneous".

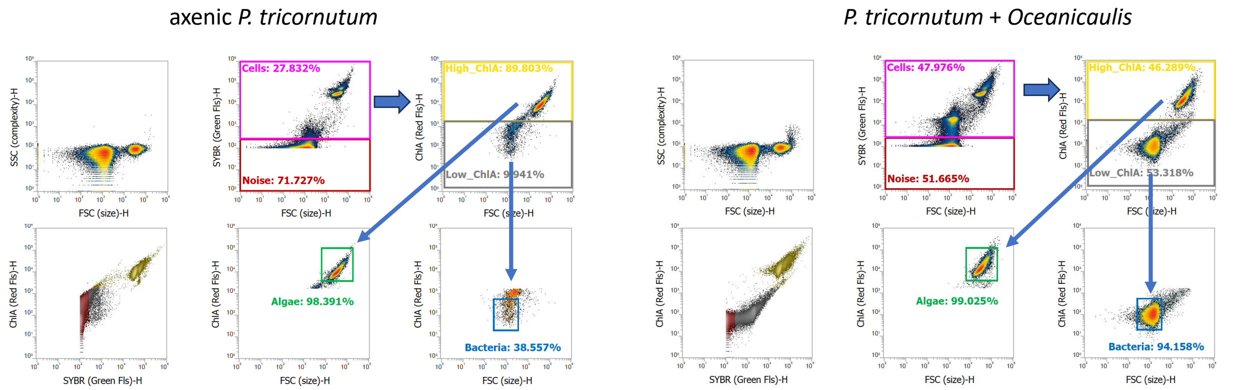

Figure S13: flow cytometry cytograms for axenic Phaeodactylum (left) and a co-culture with bacteria Oceanicaulis (right). Green fluorescence and forward scatter is first used to remove noise and then bacteria are separated from algae based on red fluorescence and forward scatter.
