# Supplementary material for: Single-cell isotope tracing reveals functional guilds of bacteria associated with the diatom Phaeodactylum tricornutum
Source: Nat Commun. 2023 Sep 13;14:5642. doi: 10.1038/s41467-023-41179-9 (PMC10499878; doi:10.1038/s41467-023-41179-9)
Supplement: Supplementary file 3 — Description of Additional Supplementary Files [file 41467_2023_41179_MOESM3_ESM.pdf]

Description of Additional Supplementary Files:

Supplementary Data 1: expansion of Table 1, which includes calculated bacterial biovolumes and C and N content

Supplementary Data 2: results of proteomics showing proteins expressed by Marinobacter and Rhodophyticola in co-culture with Phaeodactylum tricornutum

Supplementary Data 3: results of genomic analysis showing presence of metabolic pathways
